# Detecting the Common and Individual Effects of Rare Variants on Quantitative Traits by Using Extreme Phenotype Sampling

**DOI:** 10.3390/genes7010002

**Published:** 2016-01-14

**Authors:** Ya-Jing Zhou, Yong Wang, Li-Li Chen

**Affiliations:** 1 Department of Mathematics, School of Science, Harbin Institute of Technology, Harbin 150001, China; zhouyajing_0321@163.com (Y.-J.Z.); chenlili_02_06@163.com (L.-L.C.); 2 School of Mathematical Sciences, Heilongjiang University, Harbin 150080, China

**Keywords:** association study, extreme sampling, random sampling, rare variants

## Abstract

Next-generation sequencing technology has made it possible to detect rare genetic variants associated with complex human traits. In recent literature, various methods specifically designed for rare variants are proposed. These tests can be broadly classified into burden and nonburden tests. In this paper, we take advantage of the burden and nonburden tests, and consider the common effect and the individual deviations from the common effect. To achieve robustness, we use two methods of combining *p*-values, Fisher’s method and the minimum-*p* method. In rare variant association studies, to improve the power of the tests, we explore the advantage of the extreme phenotype sampling. At first, we dichotomize the continuous phenotypes before analysis, and the two extremes are treated as two different groups representing a dichotomous phenotype. We next compare the powers of several methods based on extreme phenotype sampling and random sampling. Extensive simulation studies show that our proposed methods by using extreme phenotype sampling are the most powerful or very close to the most powerful one in various settings of true models when the same sample size is used.

## 1. Introduction

Hundreds of common genetic variants associated with many complex diseases and human traits have been successfully identified by the genome-wide association studies (GWAS). However, these common genetic variants with minor allele frequencies (MAF ) >3% have small to moderate effects, and explain only a small fraction of disease heritability for common disease [1,2,3,4]. Thus, it has been hypothesized that rare variants with MAF <3% may account for some of the missing hereditability [5,6,7,8,9,10]. Next-generation sequencing technology will soon sequence the whole genome of large groups of individuals and thus will make testing rare variants possible. Unfortunately, rare variants are difficult to detect even with large sample size. Thus, we need to develop powerful study designs.

Various methods have been proposed to detect association between rare variants and complex diseases. These tests can be broadly classified into burden and nonburden tests. Burden tests collapse multiple rare variants in a genetic region into a single variant, and then test the association between the single variant and the trait of interest. Burden tests include the cohort allelic sums test (CAST) [11], the combined multivariate and collapsing (CMC) method [12], the weighted methods [13], and the variable minor allele frequency threshold method [14]. The same strategy is used in many methods [15,16,17,18]. In fact, burden tests detect the common effect of all rare variants in a region. Thus, burden tests are powerful when the effects of all rare variants in a region are in the same direction and all variants are causal variants. However, these tests will suffer great power loss when these assumptions are violated. Nonburden tests, which are called “variance component tests”, use the kernel machine regression framework. In this framework, the effects of variants are assumed to be independently and identically distributed with a mean 0 and variance $\tau^{2}.$ To test whether a set of variants is associated with the phenotype, it is equivalent to test whether the variance $\tau^{2}=0$. Examples of nonburden tests include C-alpha [19], the sequence kernel association test (SKAT) [20], the optimal SKAT (SKAT-O) [21], the mixed effects test (MiST) [22], and an optimally weighted combination of variants (TOW) [23]. Variance component tests are more powerful than burden tests when a genetic region has both protective and deleterious variants or many noncausal variants.

From the nonburden tests, we can see that the average association across variants is zero. However, unless the effect of all rare variants are in opposite directions with the same strength, and they cancel out, the average effect will not be zero. Thus, a model restricting the average effect to be zero may lose power. Thus, we use a more flexible model proposed by Wang *et al*. [24]. In this model, we take advantage of the burden and nonburden tests, and consider the common effect and the individual deviations from the common effect. In order to increase the power, we consider Fisher’s method and minimum-*p* method of combining *p*-values.

In this paper, we also explore the advantage of the extreme phenotype sampling in rare variant analysis and refine this design framework for future large-scale association studies on quantitative traits. Sampling individuals with extreme phenotypes can enrich the frequency of rare variants and therefore lead to an increase in power compared to random sampling. Recently, several statistical methods have been proposed for rare variants association study when extreme phenotypes are sampled [25,26,27,28,29]. Here, we use random sampling and extreme phenotype sampling, and compare the powers of different methods by the two sample techniques in the same sample size. In extreme phenotype sampling, we sample the individuals with higher trait value as cases and sample the individuals with lower trait value as controls. A logistic model is used for these “case-control” data. We conduct a large number of simulations and then analyze the type I error rates and powers of several methods.

## 2. Materials and Methods

### 2.1. Materials

We only consider the quantitative traits. Consider a sample of *n* individuals and *p* rare variants in a genomic region. For i=1,2,⋯,n, let $Y_{i}$ denote the trait value of the *i*th individual; for i=1,2,⋯,n and j=1,2,⋯,p, let $G_{ij}$ denote the number of the minor alleles that the *i*th subject carries at the *j*th variant site.

### 2.2. Methods

Consider a linear regression model

(1) $$Y_{i}=\beta_{0}+\beta_{1}G_{i1}+\cdots +\beta_{p}G_{ip}+\varepsilon_{i},$$

where $\beta ={\left(\beta_{1},\beta_{2},\cdots ,\beta_{p}\right)}^{\prime }$ is the regression coefficients for $G_{i}={\left(G_{i1},G_{i2},\cdots ,G_{ip}\right)}^{\prime }$, $\varepsilon_{i}$ is an error term with a mean of zero and a variance of $\sigma^{2}.$ Testing whether there is effect of a set of rare variants on a trait is equivalent to testing the null hypothesis $H_{0}:\beta =0,$ that is, $\beta_{1}=\beta_{2}=\cdots =\beta_{p}=0.$ For rare variants, the likelihood ratio test with *p* degrees of freedom has low power.

To decrease degrees of freedom and increase the power, burden test collapses the *p* rare variants into a single variant. Then, the model is simplified as

(2) $$Y_{i}=\theta_{0}+\theta_{1}C_{i}+\varepsilon_{i},$$

where $C_{i}=\sum_{j=1}^{p}G_{ij}$, $\theta_{1}$ represents the common effect across all rare variants. The null hypothesis of no association is $H_{0}:\theta_{1}=0.$ Burden score test statistic of $\theta_{1}=0$ is

(3) $$T_{B}={\left(\frac{1}{\sigma^{2}}\sum_{i=1}^{n}C_{i}\left(Y_{i}-\bar{Y}\right)\right)}^{2},$$

where $\bar{Y}=\frac{1}{n}\sum_{i=1}^{n}Y_{i}$, and $\sigma^{2}$ is estimated by the variance of *Y*. When these individuals are randomly sampled, the burden test is denoted as RS_burden.

In practice, it may be more likely that the very extremes of phenotype distribution may consist of unknown genetic heterogeneity due to genes with large effects (*i.e.*, Mendelian disorders). In such cases, the corresponding variants will be enriched in the extreme sample. Thus, we think that extreme phenotype sampling will be more powerful than random sampling. For example, in clinical practice, diseases such as hypertension and obesity, are dichotomized by setting a threshold for quantitative traits. When individuals with extreme phenotypes are sampled, the high phenotypic extremes are regarded as cases and the low phenotypic extremes are regarded as controls. The logistic model for these “case-control” data is

(4) $$\mathrm{logit}P\left(Y_{i}=1\right)=\theta_{0}+\theta_{1}C_{i}.$$

The test statistic of $H_{0}:\theta_{1}=0$ is

(5) $$T_{B}^{\prime }={\left(\sum_{i=1}^{n}C_{i}\left(Y_{i}-\bar{Y}\right)\right)}^{2}.$$

This method of using extreme phenotype sampling is denoted as ES_burden. We note that the two models assume that all rare variants have the same magnitude and the same direction effects on the phenotypes. When these assumptions are violated, the two methods can suffer from power loss.

Here, we use the following model [24]

(6) $$Y_{i}=\theta_{0}+\theta_{1}C_{i}+\beta_{1}G_{i1}+\cdots +\beta_{p}G_{ip}+\varepsilon_{i},\;E\left(\beta_{j}\right)=0,\mathrm{cov}\left(\beta \right)=\sigma_{\beta }^{2}I_{p},\;\varepsilon_{i}∼N\left(0,\sigma^{2}\right),$$

where $\theta_{1}$ represents the common effect across all rare variants and is regarded as fixed effect, $\beta ={\left(\beta_{1},\beta_{2},\cdots ,\beta_{p}\right)}^{\prime }$ represents the vector of individual effect deviations from the common effect and is regarded as random effect. Under this model, testing whether the rare variants influence the phenotype corresponds to testing the null hypothesis

(7) $$H_{0}:\theta_{1}=0,\sigma_{\beta }^{2}=0.$$

In the Appendix, we show that the score statistic for the null hypothesis $H_{0}$ is given by

(8) $$S={\left(S_{1},S_{2}\right)}^{\prime }={\left(\frac{1}{\sigma^{2}}\sum_{i=1}^{n}C_{i}\left(Y_{i}-\bar{Y}\right),\frac{1}{2}U^{\prime }U-\frac{1}{2\sigma^{2}}tr\left(G^{\prime }G\right)\right)}^{\prime }$$

where $U={\left(U_{1},\cdots ,U_{p}\right)}^{\prime }$, $U_{k}=\frac{1}{\sigma^{2}}\sum_{i=1}^{n}G_{ik}\left(Y_{i}-\bar{Y}\right)$ and $G={\left(G_{1},G_{2},\cdots ,G_{n}\right)}^{\prime }.$ Let $p_{1}$ denote the two-sided *p*-value of $S_{1}$, and $p_{2}$ denote the *p*-value of $S_{2}.$ To combine the *p*-values obtained from $S_{1}$ and $S_{2}$, we propose to use Fisher’s method, and the test statistic of $H_{0}:\theta_{1}=0,\sigma_{\beta }^{2}=0$ is defined as

(9) $$T_{1}=-2\log p_{1}-2\log p_{2}.$$

In addition, we consider other methods for combining *p*-values, such as the minimum-*p* approach. In the minimum-*p* approach, the test statistic of $H_{0}$ is defined as

(10) $$T_{2}=\min\left\{p_{1},p_{2}\right\}.$$

The two methods of combining P-values have been studied by many authors [22,30]. When we consider random sampling, the two methods are respectively called RS_Fisher and RS_min-p.

Next, we consider extreme phenotype sampling. Dichotomizing the higher and the lower phenotypic extremes as cases and controls, the logistic regression model for these “case-control” data is

(11) $$\mathrm{logit}\left\{P\left(Y_{i}=1\right)\right\}=\theta_{0}+\theta_{1}C_{i}+\beta_{1}G_{i1}+\cdots +\beta_{p}G_{ip},\;E\left(\beta_{j}\right)=0,\mathrm{cov}\left(\beta \right)=\sigma_{\beta }^{2}I_{p}.$$

In the Appendix, we show that the score statistic for the null hypothesis $H_{0}:\theta_{1}=0,\sigma_{\beta }^{2}=0$ is

(12) $$S={\left(S_{1},S_{2}\right)}^{\prime }={\left(\sum_{i=1}^{n}\left(Y_{i}-\bar{Y}\right)C_{i},\frac{1}{2}U^{\prime }U-\frac{1}{2}tr\left(\bar{Y}\left(1-\bar{Y}\right)G^{\prime }G\right)\right)}^{\prime },$$

where $U={\left(U_{1},\cdots ,U_{p}\right)}^{\prime }$ and $U_{k}=\sum_{i=1}^{n}G_{ik}\left(Y_{i}-\bar{Y}\right).$ $p_{3}$ and $p_{4}$ are respectively the *p*-values obtained from $S_{1}$ and $S_{2}$. We also use the two methods of combining *p*-values: Fisher’s method and minimum-*p* method. The test statistics of $H_{0}:\theta_{1}=0,\sigma_{\beta }^{2}=0$ is

(13) $$T_{3}=-2\log p_{3}-2\log p_{4},\;\;\;\mathrm{or}\;\;\;T_{4}=\min\left\{p_{3},p_{4}\right\}.$$

We denote the two methods as ES_Fisher and ES_min-p, respectively. We use permutation approach to obtain the *p*-value of the statistic $T_{j}$, for j=1,2,3,4. The permutation process is the same as that of Lin *et al*. [31].

## 3. Simulation and Results

### 3.1. Simulation Design

The GAW17 provides the Mini-Exome genotype data for simulation studies. This dataset contains genotypes and phenotypes for 697 unrelated individuals on 3205 genes. We follow the simulation set-up of Sha *et al*. [23]. Specifically, we select a gene (ADAMTS4) with 40 variants, and infer its haplotypic phases for the 697 individuals. To generate the genotypes with 40 variants for *N* individuals, we randomly combine two haplotypes of the 697 individuals.

To evaluate type I error rate, we generate quantitative trait values by using the model:

(14) $$Y_{i}=\beta_{0}+\varepsilon_{i},$$

where $\beta_{0}=0.1$, $\varepsilon_{i}$ follows a standard normal distribution. We estimate the empirical type I error rate as the proportion of *p*-values less than α=0.01 or 0.05.

To evaluate power, we generate phenotypes for the *N* individuals by using the following model:

(15) $$Y_{i}=\beta_{0}+\beta_{1}G_{i1}+\beta_{2}G_{i2}+\cdots +\beta_{p}G_{ip}+\varepsilon_{i},$$

where $\beta_{0}=0.1$, $\varepsilon_{i}$ follows a standard normal distribution. Effects of causal variants depend on minor allele frequencies (MAF), *i.e.*, $|\beta_{j}|=-0.2\log_{10}\left({\mathrm{MAF}}_{j}\right).$ The percentages of causal variants with MAF <0.03 are assigned three values: 40%, 60%, and 80%. The percentages of causal variants with positive effect are assigned three values: 50%, 80%, and 100%. We also consider different sample sizes (*n* = 500, 1000, and 2000) and the proportions of extreme phenotypes (10% and 20%).

After the genotype and phenotype data for *N* individuals are simulated, for random sampling, *n* individuals are arbitrarily selected from the *N* individuals. For extreme phenotype sampling, we denote the highest n/2 extremes from the *N* individuals as cases and the lowest n/2 extremes as controls. The method of Wang *et al.* [24] is denoted as JOINT. The RS_Fisher, the RS_min-p, the RS_burden and the JOINT use random sampling, the ES_Fisher, the ES_min-p, and the ES_burden use extreme phenotype sampling.

### 3.2. Evaluation on Type I Error Rates

For type I error rates, we consider different sample sizes, different proportions of extreme phenotypes, and different significance levels. In each simulation setting, *p*-values are estimated by 500 permutations and type I error rates are evaluated by 1000 replications. The estimated type I error rates of the seven methods (JOINT, RS_Fisher, RS_min-p, ES_Fisher, ES_min-p, RS_burden and ES_burden) are summarized in Table 1. From Table 1, we can see that the estimated type I error rates are not significantly different from the nominal levels. Thus, all tests are valid tests.

**Table 1:** The estimated type I error rates for all tests.

Tails	Sample Size	*α*	JOINT	RS_Fisher	RS_min-p	ES_Fisher	ES_min-p	RS_Burden	ES_Burden
0.1	500	0.01	0.015	0.013	0.013	0.014	0.013	0.010	0.016
	1000	0.01	0.011	0.010	0.018	0.008	0.003	0.015	0.008
	2000	0.01	0.011	0.012	0.012	0.011	0.013	0.012	0.016
	500	0.05	0.047	0.051	0.045	0.043	0.043	0.048	0.047
	1000	0.05	0.057	0.048	0.052	0.050	0.052	0.042	0.047
	2000	0.05	0.050	0.049	0.050	0.050	0.050	0.049	0.052
0.2	500	0.01	0.008	0.005	0.007	0.009	0.011	0.009	0.015
	1000	0.01	0.012	0.012	0.012	0.011	0.010	0.010	0.013
	2000	0.01	0.009	0.013	0.013	0.014	0.011	0.018	0.018
	500	0.05	0.053	0.049	0.054	0.041	0.044	0.054	0.037
	1000	0.05	0.046	0.040	0.039	0.052	0.061	0.036	0.052
	2000	0.05	0.041	0.049	0.052	0.050	0.045	0.050	0.049

Note: “tails” represents 10% or 20% high/low extreme phenotype sampling; *α* represents the significance level.

### 3.3. Power Comparisons

For power comparisons, we consider different sample sizes, different proportions of extreme phenotypes, different percentages of causal variants, and different percentages of causal variants with positive effects. In each simulation scenario, *p*-values are estimated by 500 permutations and powers are evaluated using 500 replications at a significance level of 0.05. In all cases, the threshold value of rare variants is selected as 0.03.

**Figure 1:**
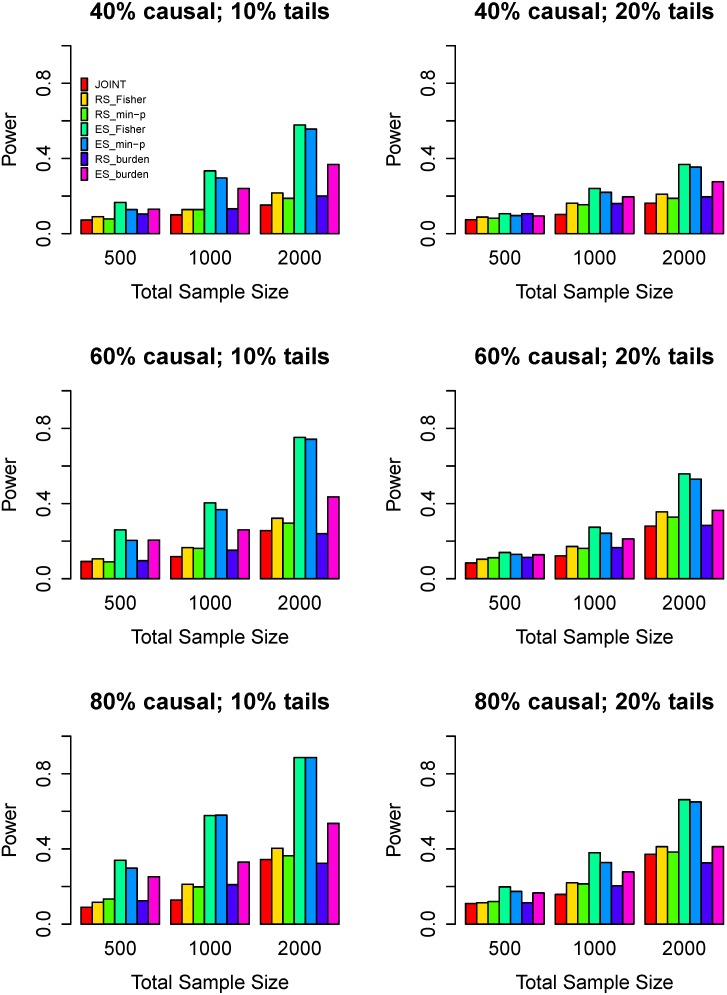
Power comparisons of seven tests when 50% causal variants have a positive effect on phenotype while the remaining 50% have a negative effect. The left panel considers 10% high/low extreme phenotype sampling with the three rows corresponding to 40%, 60%, and 80% causal variants. The right panel considers 20% high/low extreme phenotype sampling. Three sample sizes are considered: *n* = 500, 1000, 2000. Powers are estimated at the 0.05 significance level.

Power comparisons of the seven tests for half risk variants and half protective variants are given in Figure 1. As shown in Figure 1, the three tests with extreme phenotype sampling are more powerful than the other four tests with random sampling. The ES_Fisher and the ES_min-p have similar powers, and they are much more powerful than the ES_burden. Among the other four tests (JOINT, RS_Fisher, RS_min-p, and RS_burden), the JOINT is the least powerful one. The RS_Fisher and RS_min-p are slightly better than the RS_burden. The powers of all tests increase with the increase of the sample size. All tests show an increase in power with the increase of the percentage of causal variants given the same sample size. In particular, as the percentage of extreme sample increases from 10% to 20%, the powers of all tests decrease, but the power difference among all tests reduces. This is because a big percentage of extreme sampling is similar to the random sampling, which makes minor allele frequencies decrease, so that the test powers suffer loss.

**Figure 2:**
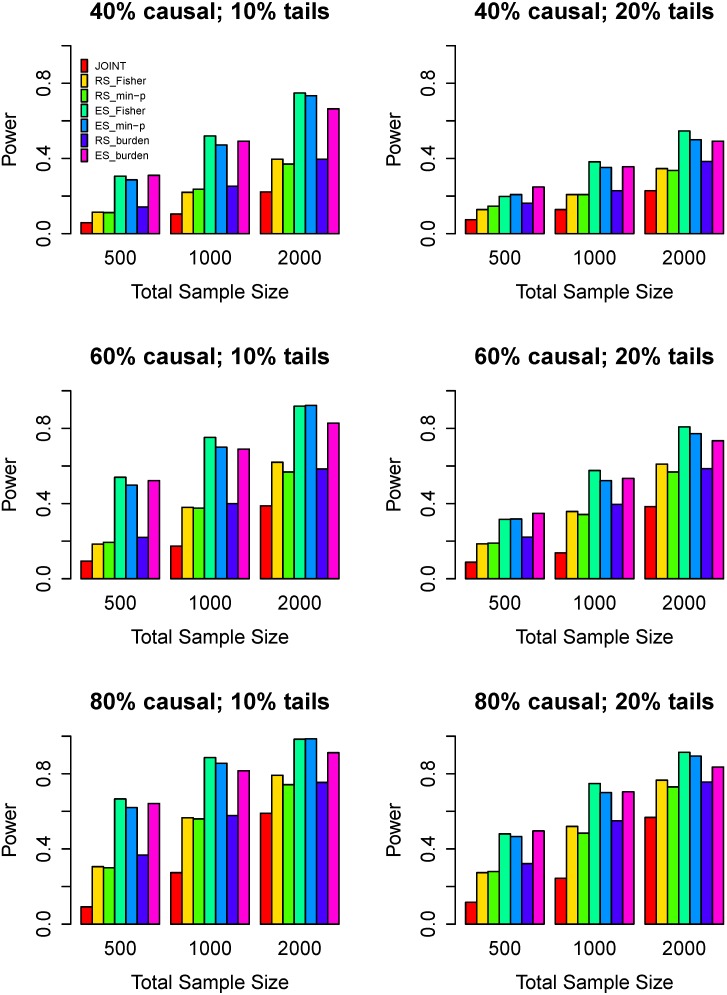
Power comparisons of seven tests when 80% causal variants have a positive effect on phenotype while the remaining 20% have a negative effect. The left panel considers 10% high/low extreme phenotype sampling with the three rows corresponding to 40%, 60%, and 80% causal variants. The right panel considers 20% high/low extreme phenotype sampling. Three sample sizes are considered: *n* = 500, 1000, 2000. Powers are estimated at the 0.05 significance level.

Power comparisons of the seven tests for 80% risk variants and 20% protective variants are given in Figure 2. By comparing Figure 2 with Figure 1, we can see that the powers of all tests increase uniformly and patterns of power comparisons is very similar. The difference of the ES_Fisher and the ES_burden decreases.

**Figure 3:**
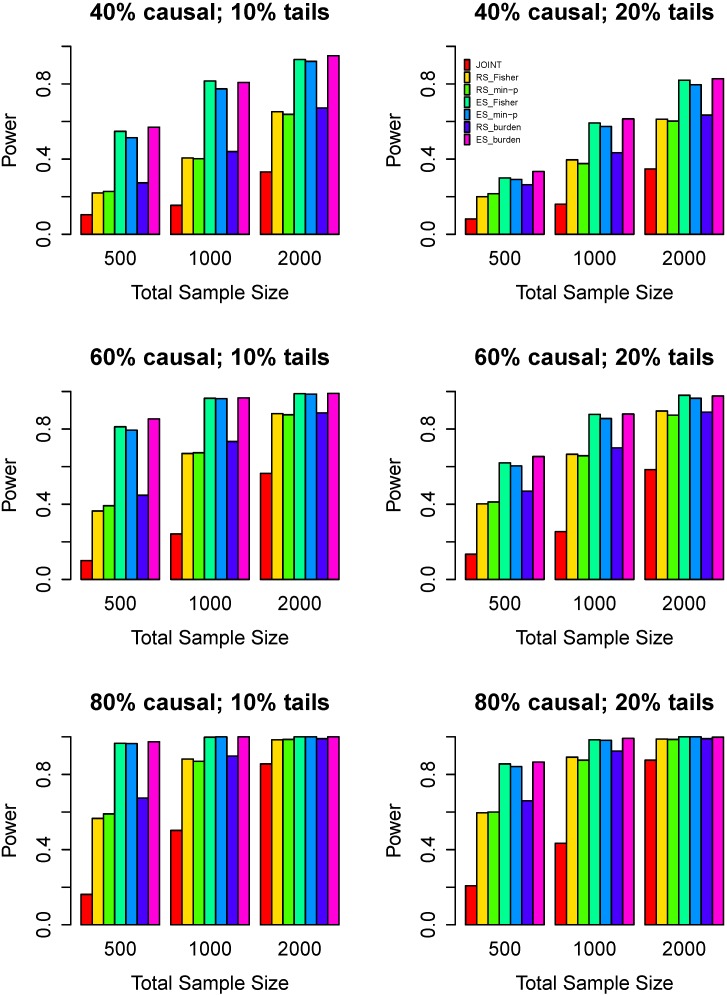
Power comparisons of seven tests when all causal variants have the same effect direction. The left panel considers 10% high/low extreme phenotype sampling with the three rows corresponding to 40%, 60%, and 80% causal variants. The right panel considers 20% high/low extreme phenotype sampling. Three sample sizes are considered: *n*=500, 1000, 2000. Powers are estimated at the 0.05 significance level.

Power comparisons of the seven tests for the same direction effect of variants are given in Figure 3. The ES_Fisher, the ES_min-p and the ES_burden have similar powers. The ES_burden are slightly better than the ES_Fisher. From Figure 1, Figure 2 and Figure 3, we can see that the difference of the ES_Fisher and the ES_burden decreases gradually. This is because the burden tests assume that variants have the same direction effects and all variants are causal, but our proposed methods allow for different direction effects of variants and also allow for the inclusion of noncausal variants. Thus, when risk and protective variants are present, the burden tests suffer substantial loss of power.

In summary, the ES_Fisher and the ES_min-p are either the most powerful tests or have similar powers to the most powerful one in each setting. The powers of the ES_Fisher and the ES_min-p are relatively robust to the increase of protective variants and neutral variants. It means that in rare variants association studies, extreme phenotype sampling is superior to random sampling in the same sample size.

## 4. Discussion

GWAS have identified many genetic variants associated with many multifactorial diseases. However, most GWAS approaches do not consider the disease heterogeneity and the follow up functional analysis of risk variants. Recently, a new field of ‘molecular pathological epidemiology (MPE)’ has emerged as an interdisciplinary integration of ‘molecular pathology’ and epidemiology” [32]. The MPE research approach mainly examines the relationships between potential etiological factors and disease subtypes based on molecular signatures [33]. In addition, MPE also assesses the interactive effects of environmental influences and disease molecular signatures on disease progression. MPE can be one of the next steps of GWAS. Thus, the GWAS-MPE approach was proposed, to take disease heterogeneity into account following GWAS analyses [34]. In the traditional GWAS, a disease of interest is regarded as a single entity without consideration of heterogeneity. By employing the MPE approach, molecular disease classification can help to identify a specific disease subtype that is more strongly associated with a given risk variant than other subtypes of the same disease. A basic approach of MPE is a case-case approach, where diseases are classified into subtypes according to a molecular feature and then distributions of an exposure variable of interest among different subtypes are compared. Thus, in this paper, we may classify into subtypes according to a molecular feature, and then compare the distributions of an exposure variable of interest among different subtypes. We may also examine how lifestyle or genetic factors interact with the molecular features to influence prognosis or clinical outcome. This is something the authors are working on for the future.

The idea of sampling the extremes was initially proposed in linkage analysis as a way to increase efficiency [35]. However, the potential gain by sampling the extremes and technical details of this design has not been well established. For planning future large-scale association studies, we explored the advantage of extreme phenotype sampling for rare variants. In fact, Li *et al.* [28] have demonstrated the potential cost advantages of this design. In this paper, we have demonstrated that with the higher information content in the extreme sample, the performance of our proposed methods can be substantially improved in comparison with traditional designs. While clear advantages exist in applying extreme phenotype sampling for a quantitative trait, the realization of such advantages depends greatly on the underlying diseases mechanism. However, cancer or cardiovascular disease might have a more complex underlying mechanism, the use of extreme phenotype sampling may be limited, and the investigators need to evaluate the appropriateness of using underlying quantitative traits as a proxy for these disease mechanisms.

Our proposed methods easily adjust for covariates, such as age, gender, and principal components for population stratifications. When considering covariates, we use the following model

(16) $$g\left(E\left(Y_{i}\right)\right)=\theta_{0}+\theta_{1}C_{i}+\beta^{\prime }G_{i}+\alpha^{\prime }X_{i},$$

where $G_{i}={\left(G_{i1},G_{i2},\cdots ,G_{ip}\right)}^{\prime }$ and $X_{i}={\left(X_{i1},X_{i2},\cdots ,X_{im}\right)}^{\prime }$ are respectively the genotype and covariate of the *i*th subject. g(·) is a link function: $g\left(P\left(Y_{i}=1\right)\right)=\log\left\{P\left(Y_{i}=1\right)/P\left(Y_{i}=0\right)\right\}$ for extreme phenotype sampling; $g\left(E\left(Y_{i}\right)\right)=E\left(Y_{i}\right)$ for random sampling.

## 5. Conclusions

In this paper, we propose two methods for testing whether a set of variants is associated with continuous phenotypes. We use the same model with the JOINT method, in which common effects of all rare variants and individual effect deviations from the common effect are jointly considered. However, the SKAT assumes that the average effect is zero. In fact, the average effect will not be zero unless the effects of all rare variants are in opposite directions with the same strength.

Compared with Fisher’s method and the minimum-*p* method, the JOINT method is the sum of standardized $S_{1}$ and standardized $S_{2}$, but the Fisher’s method and the minimum-*p* method combine the *p*-values of $S_{1}$ and $S_{2}$. So the Fisher’s and minimum-*p* methods are more powerful when only all rare variants have common effect on the trait or when only rare variants have individual effects on the trait. When the true underlying disease model includes risk variants and protective variants, the Fisher’s and minimum-*p* methods are more powerful than burden tests. In the same sample size, each of the three methods (Fisher, minimum-*p*, and burden) uses random sampling and extreme phenotype sampling. Our simulation results show that sampling from extreme phenotypes outperforms random sampling methods when the same size is used.

## Appendix. Score Vector

Use the notation in the Methods section. Under the linear model

(A1) $$Y_{i}=\theta_{0}+\theta_{1}C_{i}+\beta_{1}G_{i1}+\cdots +\beta_{p}G_{ip}+\varepsilon_{i},\;E\left(\beta_{j}\right)=0,\mathrm{cov}\left(\beta \right)=\sigma_{\beta }^{2}I_{p},\;\varepsilon_{i}∼N\left(0,\sigma^{2}\right),$$

the log-likelihood is given by

(A2) $$\log L=-\frac{n}{2}\log\left(2\pi \sigma^{2}\right)-\frac{1}{2\sigma^{2}}\sum_{i=1}^{n}{\left(Y_{i}-\theta_{0}-\theta_{1}C_{i}-\beta_{1}G_{i1}-\cdots -\beta_{p}G_{ip}\right)}^{2}.$$

Then,

(A3) $$\frac{\partial \log L}{\partial \theta_{1}}=\frac{1}{\sigma^{2}}\sum_{i=1}^{n}C_{i}\left(Y_{i}-\theta_{0}-\theta_{1}C_{i}-\beta_{1}G_{i1}-\cdots -\beta_{p}G_{ip}\right),\;\frac{\partial \log L}{\partial \beta_{j}}=\frac{1}{\sigma^{2}}\sum_{i=1}^{n}G_{ij}\left(Y_{i}-\theta_{0}-\theta_{1}C_{i}-\beta_{1}G_{i1}-\cdots -\beta_{p}G_{ip}\right),\;\frac{\partial^{2}\log L}{\partial \beta_{j}\partial \beta_{l}}=-\frac{1}{\sigma^{2}}\sum_{i=1}^{n}G_{ij}G_{il}.$$

Let $\hat{\theta_{0}}$ and $\hat{\sigma^{2}}$ denote the maximum likelihood estimates of $\theta_{0}$ and $\sigma^{2}$ under null hypothesis $H_{0}:\theta_{1}=0,\sigma_{\beta }^{2}=0.$ Then, $\hat{\theta_{0}}=\bar{Y},\;\;\hat{\sigma^{2}}=\frac{1}{n}\sum_{i=1}^{n}{\left(Y_{i}-\bar{Y}\right)}^{2}.$

Using the results in Lemma 3 of Goeman *et al.* [36], we obtain

(A4) $$\frac{\partial \log L}{\partial \sigma_{\beta }^{2}}{|}_{H_{0}}=\frac{1}{2}U^{\prime }U-\frac{1}{2}tr\left(I\right)$$

where

(A5) $$U=\frac{\partial \log L}{\partial \beta }{|}_{H_{0}}=\frac{1}{\hat{\sigma^{2}}}{\left(\sum_{i=1}^{n}G_{i1}\left(Y_{i}-\bar{Y}\right),\cdots ,\sum_{i=1}^{n}G_{ip}\left(Y_{i}-\bar{Y}\right)\right)}^{\prime },\;I=-\frac{\partial^{2}\log L}{\partial \beta \partial \beta^{\prime }}{|}_{H_{0}}=\frac{1}{\hat{\sigma^{2}}}G^{\prime }G.$$

So the score vector of $H_{0}$ is

(A6) $$S={\left(S_{1},S_{2}\right)}^{\prime }={\left(\frac{\partial \log L}{\partial \theta_{1}},\frac{\partial \log L}{\partial \sigma_{\beta }^{2}}\right)}^{\prime }{|}_{H_{0}}={\left(\frac{1}{\hat{\sigma^{2}}}\sum_{i=1}^{n}C_{i}\left(Y_{i}-\bar{Y}\right),\frac{1}{2}U^{\prime }U-\frac{1}{2\hat{\sigma^{2}}}tr\left(G^{\prime }G\right)\right)}^{\prime }.$$

The score vector under the logistic model is similar to the score vector under the linear model.
